# Clinical Perspective on Proteomic and Glycomic Biomarkers for Diagnosis, Prognosis, and Prediction of Pancreatic Cancer

**DOI:** 10.3390/ijms22052655

**Published:** 2021-03-06

**Authors:** Randa G. Hanna-Sawires, Jorinde H. Schiphuis, Manfred Wuhrer, Hans F. A. Vasen, Monique E. van Leerdam, Bert A. Bonsing, Wilma E. Mesker, Yuri E. M. van der Burgt, Rob A. E. M. Tollenaar

**Affiliations:** 1 Department of Surgery, Leiden University Medical Center, 2300 RC Leiden, The Netherlands; R.G.Hanna-Sawires@lumc.nl (R.G.H.-S.); J.H.Schiphuis@lumc.nl (J.H.S.); b.a.bonsing@lumc.nl (B.A.B.); w.e.mesker@lumc.nl (W.E.M.); R.A.E.M.Tollenaar@lumc.nl (R.A.E.M.T.); 2 Center of Proteomics and Metabolomics, Leiden University Medical Center, 2300 RC Leiden, The Netherlands; m.wuhrer@lumc.nl; 3 Department of Gastroenterology, Leiden University Medical Center, 2300 RC Leiden, The Netherlands; h.f.vasen@lumc.nl (H.F.A.V.); M.E.van_Leerdam@lumc.nl (M.E.v.L.)

**Keywords:** pancreatic cancer, biomarker, mass spectrometry, proteomics, glycomics, glycosylation, early detection, prediction, prognosis, CA 19-9

## Abstract

Pancreatic ductal adenocarcinoma (PDAC) is known as a highly aggressive malignant disease. Prognosis for patients is notoriously poor, despite improvements in surgical techniques and new (neo)adjuvant chemotherapy regimens. Early detection of PDAC may increase the overall survival. It is furthermore foreseen that precision medicine will provide improved prognostic stratification and prediction of therapeutic response. In this review, omics-based discovery efforts are presented that aim for novel diagnostic and prognostic biomarkers of PDAC. For this purpose, we systematically evaluated the literature published between 1999 and 2020 with a focus on protein- and protein-glycosylation biomarkers in pancreatic cancer patients. Besides genomic and transcriptomic approaches, mass spectrometry (MS)-based proteomics and glycomics of blood- and tissue-derived samples from PDAC patients have yielded new candidates with biomarker potential. However, for reasons discussed in this review, the validation and clinical translation of these candidate markers has not been successful. Consequently, there has been a change of mindset from initial efforts to identify new unimarkers into the current hypothesis that a combination of biomarkers better suits a diagnostic or prognostic panel. With continuing development of current research methods and available techniques combined with careful study designs, new biomarkers could contribute to improved detection, prognosis, and prediction of pancreatic cancer.

## 1. Introduction

**Figure 1:**
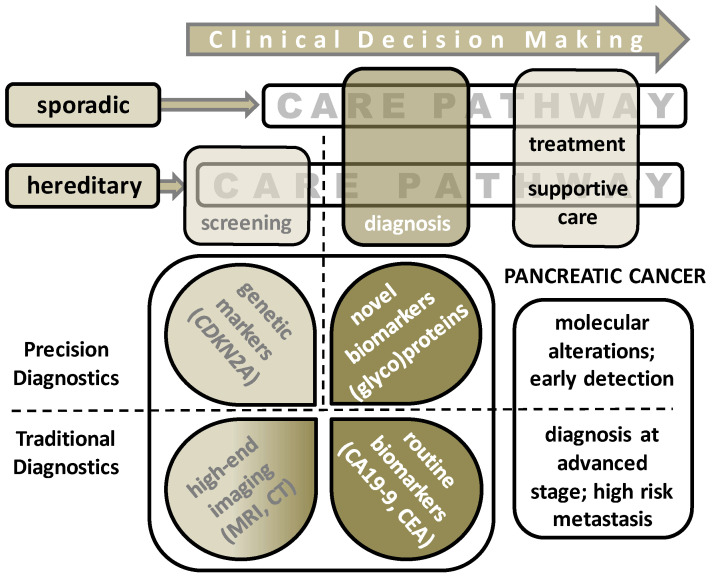
Clinical decision making pathway depicted for sporadic pancreatic ductal adenocarcinoma (PDAC) patients and individuals with a hereditary background (family history or gene mutation carriers). Surveillance of the latter group was performed with high-end imaging approaches such as MRI and EUS. Diagnosis of PDAC is commonly based on the same imaging technology combined with current routine markers, with the aid of cytology and histology diagnostics as is discussed in this review. Various ongoing approaches are summarized that search for novel (glyco)protein biomarkers.

In the previous decade, the increase in pancreatic ductal adenocarcinoma (PDAC) incidences has been higher than for other cancers. Currently, PDAC is the 11th most common cancer in the world [1]. Moreover, PDAC diagnosis implies one of the most unfavorable prognoses with a five year overall survival rate between 5–8% as a result of its aggressive tumor behavior with extensive local and metastatic spread. Often PDACs are in advanced stage upon diagnosis (80%) and curative treatment is no longer possible [2]. Available intensive treatment regimens with chemo(radio)therapy and/or surgery is associated with severe complications and side effects, resulting in an impaired quality of life [3]. Understanding tumor biology and knowledge on correlations between clinicopathologic parameters and progress of disease could provide leads for precision medicine. For example, response to therapy can be anticipated and support a decision to, in some cases, alternative options for treatment [4]. Early detection of PDAC provides a window of opportunity where treatment with curative intention is possible [5]. So far, only CA 19-9 is routinely applied as a tumor marker. However, its clinical value is limited to post-treatment follow-up and surveillance [6]. The Dutch Nationwide Program for the detection of pancreatic cancer in high-risk individuals recently reported that screening by means of imaging is insufficient and that biomarker research is warranted [7]. The clinical decision pathway for sporadic PDAC patients as well as for individuals with a hereditary risk is depicted in Figure 1. Within this care pathway, an urgent need for novel biomarkers for screening and diagnostic purposes is broadly acknowledged, and consequently, numerous exploratory studies have been performed following different methods. Better understanding of the natural course of PDAC might allow improvements in current surveillance protocols, thereby possibly improving oncological outcome. Therefore, biomarkers that can provide this information could be of great clinical benefit. An alternative method for current screening modalities could be reliable and specific biomarkers detectable in peripheral blood to detect PDAC in its earliest stage.

In this review, candidate biomarkers for PDAC detection, prognostication, and prediction are summarized, with emphasis on proteins and protein glycosylation. Most efforts have followed mass spectrometry (MS)-based proteomics or glycomics strategies [8,9,10,11,12,13]. The use of biomarkers in clinical pathways will be discussed and recommendations for further exploratory biomarker research are given.

### 1.1. Sporadic Pancreatic Cancer

The worldwide incidence of pancreatic cancer is estimated at 8.1 per 100,000 person-years and 6.9 deaths per 100,000 person-years [14]. There is great geographic variation in incidence, approximately 55% of all new cases are registered in well-developed countries [15]. In some scenarios, pancreatic cancer is projected to turn into one of the leading causes of cancer death by 2050 [16]. The two main types of pancreatic neoplasia are: adenocarcinoma (almost 93%) and endocrine tumors of the pancreas (5%). The vast majority of pancreatic cancer patients concerns sporadic pancreatic cancer [17].

Development of pancreatic cancer starts with the transformation of pancreatic cells into precursor lesions, followed by malignant degeneration. Typical precursor lesions of the pancreas are pancreatic intra-epithelial neoplasia (PanIN) and intraductal papillary mucinous neoplasm (IPMN). Not all precursor lesions develop into PDAC [18]. The true cause of malignant degeneration into PDAC remains unknown [19]. Multiple potential risk factors have been identified such as smoking, alcohol, obesity, diabetes, genetics, lifestyle, and chronic pancreatitis [16,20,21,22,23]. Chronic pancreatitis and various benign lesions can mimic PDAC and consequently lead to misclassification and overtreatment. It has been reported that 5–11% of patients who underwent pancreatic resection for presumed pancreatic cancer had benign lesions [24,25]. Identifying (a set of) biomarkers that could accurately differentiate between PDAC and benign lesions could therefore be of great benefit to clinicians.

Eighty percent of patients diagnosed with pancreatic cancer present with incurable disease [2]. The remaining 20% qualify for a curative resection. Even for those who undergo pancreatic surgery and achieve successful resection with clear resection margins (R0), survival remains low [26]. The 5-year survival rate for this group of patients is estimated at 10% [27]. Modern pancreatic cancer treatment regimens aim at combining different modalities: chemotherapy, radiotherapy, and surgery. Depending on diagnostic staging, various combinations of these modalities are applied. However, failure rates still remain high [28,29]. Hence, it is essential to develop novel cancer biomarkers that could provide clinicians with upfront knowledge on therapy response and prognosis, promoting personalized medicine [30].

### 1.2. Hereditary Pancreatic Cancer

Hereditary factors play a role in about 10% of all pancreatic cancers, yet in only 3%, an underlying gene defect can be found. Management protocols for patients with increased risk for familial pancreatic cancer are determined by the International Cancer of the Pancreas Screening Consortium (CAPS). CAPS defined certain groups that would benefit from surveillance [31]. The most common cancer syndrome for pancreatic cancer is Familial Atypical Multiple Mole Melanoma (FAMMM) syndrome, caused by a *CDKN2A* germline mutation. These individuals have a familial predisposition for developing cutaneous melanoma as well as PDAC [32]. Another known hereditary cancer type is the Peutz–Jeghers syndrome, an autosomal dominant disease caused by a germline mutation in the *STK11* gene. Patients with the Peutz–Jeghers syndrome are at higher risk of developing both gastrointestinal and extra-gastrointestinal malignancies (e.g., pancreas, esophageal, and breast carcinoma) [33,34]. Other known predisposition syndromes with an increased risk of PDAC are *BRCA1* and *BRCA2*; hereditary breast and ovarian cancer syndrome, Lynch syndrome, and ATM mutation syndrome [35,36]. In 85–90% of familial pancreatic cancer cases, no genetic mutation is found to explain the familial predisposition [22,37]. This group of patients is often referred to as familial pancreatic cancer (FPC) patients [38]. Overall, the lifetime risk of developing PDAC for individuals with hereditary risk (either carrying a genetic mutation or with familial predisposition) is 5–36% [17]. Pancreatic surveillance programs for such high-risk individuals may offer opportunities for the early detection of PDAC or relevant precursor lesions in individuals with a familial predisposition to PDAC [39]. Vasen et al. demonstrated that surveillance of individuals with a *CDKN2A* germline mutation that developed PDAC could more frequently be treated with curative intent (e.g., more surgical resections (75%), which resulted in an increased five years survival (24%) [17]).

### 1.3. Diagnosis of Pancreatic Ductal Adenocarcinoma (PDAC)

Current diagnostic methods consist of imaging techniques such as computed tomography (CT) and magnetic resonance imaging (MRI) often combined with endoscopic ultrasonography (EUS) and fine-needle-aspiration (FNA) to acquire pathological diagnosis. CT is generally able to evaluate possibilities for resection, whilst it has limited diagnostic accuracy (specificity 79%) [37]. MRI combined with cholangiopancreatography (MRCP), provides a sensitivity of 84% and 97% specificity for the detection of pancreatic cancer [40]. EUS can visualize the pancreas from nearby, enabling portrayal of small focal lesions. Canto and colleagues compared different surveillance modalities in a blinded setting. EUS detected 79%, whereas CT and MRI detected 13.8% and 77%, respectively [41]. For screening purposes, a combination of MRI and EUS is generally applied to detect early stage PDAC, however, the diagnostic success of surveillance programs using these modalities differs [39,42].

### 1.4. Treatment Strategies for PDAC

Pancreatic cancer treatment regimens currently consist of combinations of surgery, chemotherapy, and radiotherapy [43]. Preferred regimens differ per country, hospital, and depend on the stage of the disease. Until today, treatment with curative intent at least includes surgical resection. Additionally, (neo)adjuvant chemo(radio)therapy is given to improve survival rates [44] Depending on tumor location, there are different types of surgical procedures [45]. A pancreaticoduodenectomy (Whipple’s procedure) is performed for tumors located in the head of the pancreas whereas distal pancreatectomy is performed in the case of a tumor in the pancreas body or tail [44]. Pancreaticoduodenectomy is considered a high-risk surgical procedure. Due to centralization of surgical procedures for PDAC and ongoing improvements in perioperative care and technical focus, the mortality has decreased from 20% to 2–4% over the last years [28,46]. However, morbidity remains high (30–40%), while fast recovery is important for patients to start adjuvant chemotherapy to improve oncological outcome [28]. Up to 40% of patients do not qualify for adjuvant therapy due to surgical complications [47]. Since adjuvant chemotherapy is considered important for improving oncological outcome, interest has grown to provide chemotherapy before surgical resection (neoadjuvant therapy) in an effort to increase the percentage of patients receiving chemotherapy. In addition, neoadjuvant chemotherapy/chemoradiotherapy may also improve R0 resection rate, which in turn translates into significantly higher survival rates compared to incomplete (R1) resections [48,49,50,51,52,53,54]. The role of radiation therapy, adjuvant, or neoadjuvant, in pancreatic cancer treatment is unclear and is still the subject of research [55].

In an effort to minimize surgical morbidity and mortality, interest in minimally invasive techniques for pancreatic surgery has grown, first with the introduction of laparoscopic pancreatic resection, followed by robot-assisted pancreaticoduodenectomy. Numerous studies have shown that thus far, minimally invasive techniques do not improve oncological outcomes. They do show a shorter hospital stay in some studies, but more randomized controlled trials and prospective studies are needed to support current findings [46,48,49,50,51,52]. Thus far, minimally invasive techniques failed to show improved oncological outcomes.

Patients with PDAC not feasible for treatment with curative intent are usually offered palliative chemotherapy combined with symptom control. Current preferred palliative chemotherapy is FOLFIRINOX, a combination of oxaliplatin, irinotecan, fluorouracil, and leucovorin. It has a survival advantage compared to the previous standard treatment (gemcitabine) of 11.1 months versus 6.8 months, respectively. However, it has increased toxicity. The response rate to palliative chemotherapy is 11.8–31.8% with a median survival of eight months [56]. In conclusion, treatments for patients with PDAC, either with curative or palliative intent, come with complications/toxicity, resulting in a decreased quality of life and still provide poor oncological outcomes. Therefore, there is a need for biomarkers that provide information on prognosis of survival and prediction therapy response. This will enable effective personalized patient treatment regimens resulting in optimal oncological outcomes and prevention of unnecessary toxic treatments.

## 2. Current Biomarkers for Detection of Pancreatic Ductal Adenocarcinoma (PDAC)

The most frequently used biomarker and the most extensively evaluated marker for PDAC is carbohydrate antigen 19-9 (CA 19-9) [57]. CA 19-9 is a Lewis Antigen and belongs to the class of mucin-1 (MUC-1) proteins. It is commonly used as a biomarker for monitoring (or follow-up) purposes such as treatment response and disease recurrence As a biomarker, CA 19-9 exhibits two major limitations: (1) CA 19-9 can occasionally and transiently be elevated in patients with benign diseases, and (2) it has a poor predictive value of 72.3% [58]. In a study by Kim and colleagues, 70,940 asymptomatic patients were screened using CA 19-9 with a standard cut-off at 37 U/mL. Only four of the 1063 cases with elevated CA 19-9 had pancreatic cancer [59]. Xing and colleagues performed a meta-analysis on the diagnostic value of CA 19-9. A total of 13 studies were included and a poor specificity (68–80%) and a sensitivity of 80% was reported [60]. The accuracy of CA19-9 as a diagnostic marker is not sufficient to be used in high risk individuals or in the general population.

The second most used diagnostic biomarker for PDAC is carcinoembryonic antigen (CEA), a glycoprotein that was originally identified as a biomarker for colorectal cancer and has since been evaluated as a diagnostic marker for several cancers [61,62]. Poruk and colleagues performed a meta-analysis on CEA as a diagnostic marker for PDAC. A total of 23 studies were included. Most cases included PDAC, but benign pancreatic disease patients were also analyzed. Meta-analysis showed a mean sensitivity of 44.2% (*p* ≤ 0.001) and specificity of 84.8% (*p* = 0.29). The difference in sensitivity and specificity is explained by the different analytical technique using mean versus median estimates. In the same meta-analysis, CA19-9 had a mean sensitivity of 78.2% (*p* ≤ 0.001) and specificity of 82.8% (*p* ≤ 0.001) [57]. CA 19-9 nor CEA possess the desired accuracy in order to use these markers for screening in asymptomatic high risk or general populations [63]. In clinical practice, CA 19-9 and CEA are used in combination with other diagnostic tools as a follow-up marker or for directing treatment decisions [57].

## 3. The Pursuit of Novel Diagnostic PDAC Biomarkers

PDAC diagnosis needs to be discerned from chronic pancreatitis and other benign and (pre)malignant diseases of the pancreas to allow precise patient selection for curative surgery. However, diagnosis by CT/MRI is not perfectly specific and cytology/histology not always possible to obtain, and as a result, approximately 5–11% of the patients receive surgical overtreatment [24,25,64]. A clinical need to explore new methods for diagnosing PDAC and differentiate from benign pancreatic diseases and improve patient selection preoperatively is therefore apparent. In addition, detection of PDAC at an earlier stage increases the possibility for treatment with curative intent, which in turn might translate into improved overall survival [17,65]. A recent review on sixteen pancreatic cancer screening studies of high-risk individuals showed that early detection resulted in a higher curative resection rate (60% vs. 25%, *p* = 0.011) and a longer median survival (14.5 months vs. four months, *p* < 0.001) [66].

The need for an improved diagnostic test for pancreatic cancer is also high for patients with increased inherited risk. Such a test should meet the specific requirements and should exhibit suitable sensitivity and specificity specifications that would render screening modalities feasible. Multiple studies have been performed aiming at a test that combines CA 19-9 with other protein biomarkers. For example, Park and colleagues described the performance of CA 19-9 together with apolipoprotein A-IV and metalloproteinase-1 in a cohort of 182 individuals consisting of 42 early stage (I/II) PDAC patients, 74 advanced stage (III/IV) PDAC patients, 31 pancreatitis patients, and 35 healthy controls. The sensitivity was 86% at fixed 90% specificity versus a sensitivity of 71% for CA 19-9 alone [67]. Alternatively, Kim and colleagues used plasma samples of 81 PDAC patients and 80 healthy controls and found that elevated levels of plasma thrombospondin-2 (TSP-2) can distinguish PDAC patients from healthy controls with a sensitivity of 87% and a specificity of 98% when combined with CA 19-9 [68]. Furthermore, Liu and colleagues described a novel potential biomarker panel consisting of: apolipoprotein E (APOE), inter-alpha-trypsin inhibitor heavy chain H3 (ITIH3), apolipoprotein A-I (APOA1), and apolipoprotein L1 (APOL1), combined with CA19-9. The cohort consisted of 80 PDAC patients, 30 patients with benign pancreatic disease (e.g., pancreatitis, benign tumor), and 40 healthy controls. This panel showed a statistically significantly improved sensitivity (95%) and specificity (94.1%), outperforming CA19-9 for the diagnosis of PDAC. Despite these promising results, these studies included a limited number of cases and require further evidence to replicate and validate these results in screening settings [69]. The same is true for other exploratory studies that have been performed using retrospective clinical sample cohorts (body fluids such as plasma, serum, and pancreatic juice) in a case-control setup. Candidate markers require replication in larger sample cohorts [70,71,72,73,74,75,76]. However, such validation of candidate markers is difficult to achieve due to an often long follow up time and limitations with regard to patient consent. So far, none of the biomarker candidates have been translated into clinical practice. Whereas initially technical imprecision and complexity of the applied MS-based strategies were held responsible for this disappointing outcome, it soon became clear that the lack of a thoughtful study design and standard protocols may have influenced successful biomarker development. In addition, it should be kept in mind that tumors exhibit a heterogeneous character due to the various cell types within their microenvironment, and that clonal evolution can lead to various cellular subpopulations.

### 3.1. Biomarker Discovery Studies by Mass Spectrometry (MS)-Based Proteomics and Glycomics

**Figure 2:**
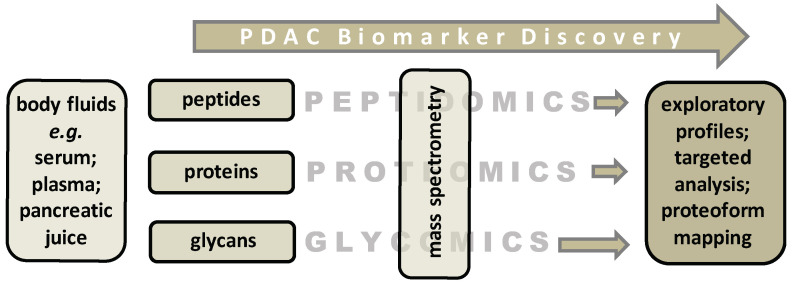
Overview of previous and ongoing PDAC biomarker discovery studies. This review has focused on three types of biomolecules that are obtained from patient body fluids. Potential biomarkers identified from tissue-based samples were out-of-scope. Without detailing the various mass spectrometry technologies, the applied strategies can either have an exploratory or a targeted nature. A third strategy, namely proteoform mapping, is explained in the main text.

Recently, we reported on N-glycome analysis that allowed differentiation of PDAC patients from healthy controls with a combination of three so-called derived glycosylation traits (antennarity, sialylation, and fucosylation with an area-under-the-curve of 0.81–0.88) [9]. This study is a first step in the pursuit of novel glycan markers that made use of earlier technological developments in proteomics. Initially, both high-end and automated analytical strategies that have been developed for MS-based proteomics purposes were applied to measure peptide and protein levels in biological samples with the analytical robustness required for clinical application [77] (summarized in Figure 2).

In these early MS-based proteomics studies, patient serum samples were used to evaluate the profiling potential of body fluids with minimal sample workup. For example, Velstra and colleagues were able to discriminate a peptide/protein signature of pancreatic cancer patients from healthy individuals using ultrahigh-resolution matrix-assisted laser desorption/ionization (MALDI) time-of-flight MS. In that study, serum samples were obtained from sporadic pancreatic cancer patients (N = 89) and healthy volunteers (N = 185) and divided into a calibration and validation set. The discriminating profile showed a sensitivity of 78% and a specificity of 89%, validated with a sensitivity of 74% and a specificity of 91% [78]. This work was continued by combining MALDI with ultrahigh resolution instrumentation that provides more detailed peptide signatures [79]. Differentiating peptides were identified from thrombin light chain, fibrinogen alpha, platelet factor 4, complement C3f, and factor XIIIa protein. This signature was further used in a study to classify hereditary PDAC cases in a surveillance cohort from control samples (discriminant score of 0.26 vs. 0.016; *p* = 0.001). All individuals had a CDKN2A germline mutation and were enrolled in the pancreatic surveillance program [80]. A combination of these peptides could be useful for future clinical applications, but replication and validation on a larger cohort is still needed.

Reproducibility of biomarker discovery studies for PDAC screening remains challenging and the availability of validated results is limited [81,82]. Therefore, the proteomics field has moved to a standardized approach that results in improved reproducibility, although it involves more work before MS-analysis. A recent study by Jiyoung Park and co-workers performed 1000 biomarker candidate research on 134 plasma samples using multiple reaction monitoring MS. From the 1000 potential biomarkers that have previously been studied, a thorough selection was made based on previous study outcomes. Only biomarker candidates that previously showed an AUC > 0.60 were selected, leaving them with 176 proteins to test. The cohort consisted of 50 PDAC patients, 34 precursor lesions patients (IPMN), and 50 healthy volunteers. Following relative quantification and triplicate analyses, 54 proteins showed a AUC > 0.60 to discriminate between PDAC and healthy volunteers. Afterward, a cross-platform validation study was performed with 1008 plasma samples to validate the previously analyzed 54 proteins. A multimarker panel was found consisting of leucine-rich alpha-2 glycoprotein (LRGI), transthyretin (TTR) and CA 19-9 with a sensitivity of 82.5% and a specificity of 92.1%. It exceeded the performance of CA 19-9 alone in that same study by 10%. The sensitivity for distinguishing surgically resectable early stage PDAC from advanced stages in this study was 64% [83]. Although these results look promising, one should consider false positive results leading to unnecessary diagnostics as well as false negative results leading to missed cases in high risk populations. Moreover, samples were obtained at five different locations (medical centers) and potential differences in pre-analysis could have an influence on the results.

Alternatively, the currently applied protein routine markers can be re-visited, that is in-depth analysis can be performed to shed light on their precise molecular structures. It is hypothesized that so-called proteoform profiles of existing (clinically relevant) markers may provide an additional structural layer to quantitative levels of individual proteins with potential for patient stratification [84,85]. In this context, protein glycosylation provides an interesting source of potential leads. Protein glycosylation is an enzymatically regulated process in which glycans covalently attach to specific amino acids in proteins. It plays an important role in many biological mechanisms such as cell adhesion, protein folding, trafficking, pathogen recognition, and immune response. At least 50% of cell proteins are found to be glycosylated [86]. The first knowledge on glycosylation and the association with cancer already dates from over 50 years ago. However, during the last decade, the field of glycobiology has evolved enormously [86,87,88,89]. The aberrant glycosylation profile on the surface of cancer cells has been found to be of potential diagnostic value toward evaluating tumor progression [90]. Mucin (MUC) proteins, often described as CA 19-9 protein carriers, are found to be of influence on pancreatic cancer tumorigenesis, invasiveness, and metastasis. However, applicability is often described to be limited due to several reasons: expression in benign diseases (e.g., pancreatitis), low predictive value in asymptomatic patients (0.5–0.9%), and varying specificity (70–90%) and sensitivity (68–91%) [91,92,93].

From an alternative body fluid material, namely extracellular vesicles, Glypican 1 and Glycoprotein 2 were detected in a study by Melo and colleagues [94]. However, the sensitivity and specificity of Glypican 1 only or Glypican 1 and Glycoprotein 2 combined was insufficient to differentiate between malignant and benign disease [95]. SPAN-1, a high molecular weight glycoprotein, was first described in 1990 as a potential diagnostic marker. Kiriyama and colleagues studied the sera of 64 PDAC patients, 90 with other types of cancers, 254 non-malignant patients, and 55 healthy controls. Even though it showed a sensitivity and specificity of 81.3% and 75.6%, respectively, it also showed high false-positive elevations in liver cirrhosis (53.8%) and chronic hepatitis (26.3%) and was therefore not clinically applicable [96]. SPAN-1 was later investigated as a potential predictive marker. Nigjeh and colleagues investigated the possible value of N-glycosylated peptides in human plasma for early detection of PDAC. They indicated that the level of N-glycosylated peptides derived from galectin-3 binding proteins (LGALS3BP) were frequently elevated in plasma from PDAC patients [97]. Krishnan and colleagues also investigated the potential of altered glycosylation of serum proteins. They provided preliminary evidence of altered glycosylation of several serum proteins (e.g., α-1-antitrypsin, haptoglobin, α-1-acid glycoprotein 1) prior to pancreatic cancer diagnosis, but stated that further investigation of these proteins as early biomarkers is indicated [98]. Nie and colleagues analyzed 179 serum samples of patients with pancreatic cancer (N = 37), chronic pancreatitis (N = 30), diabetes (N = 30), obstructive jaundice (N = 22), pancreatic cysts (N = 30), and healthy controls (N = 30). A combination of α-1-antichymotrypsin (AACT), thrombospondin-1 (THBS1), and haptoglobin (HPT) outperformed CA 19−9 in distinguishing pancreatic cancer from normal controls (AUC = 0.95), patients with diabetes (AUC = 0.89), pancreatic cysts (AUC = 0.82), and chronic pancreatitis (AUC = 0.90). However, in both studies (Krishnan et al. and Nie et al.), acute-phase proteins were used and not specific cancer-related markers [99]. Therefore, Kontro and colleagues recently studied N-glycopeptide levels in serum of PDAC and acute pancreatitis patients compared to healthy controls. An increase in sialylated N-glycopeptides was found in both PDAC and acute pancreatitis patients. Mainly N-glycopeptides derived from acute-phase proteins and immunoglobulins were found: HPT, α-1-antitrypsin (A1AT), transferrin, ceruloplasmin, α-1-acid-glycoprotein (AGP), fetuin A, and immunoglobulins [100]. The potential of glycopeptides has been further pursued in glycoproteomics studies [101]. In a recent discovery study from Aronsson and colleagues, a glycosylation profile of 1000 serum proteins was reported for eight PDAC patients and eight healthy controls. A panel including CA 19-9, IL.17E, B7.1, and DR6 showed a promising AUC of 0.988 at 100% sensitivity at 90% specificity for differentiating between PDAC and controls [102].

The described potential glycoproteins observed in patient serum could be powerful biomarkers for detection of PDAC. However, little is known on the site specific glycoproteome changes associated with PDAC. Another limitation of glycoproteins is that most of them are neither pancreatic cancer nor cancer specific [89]. To further increase specificity and improve clinical applicability, ongoing method development and validation studies are essential.

### 3.2. Prognostic Markers

There is a spectrum of biological aggressiveness of PDAC. Identification of markers that are involved in PDAC tumor progression and aggressiveness may help to select adequate treatment strategies and to retrieve knowledge on how patients will respond to therapy and in some cases perform in the absence of therapy [103]. Numerous biomarker studies have been performed over the years with the aim to discover potential prognostic biomarkers for different types of cancer (e.g., colorectal, ovarian, and breast cancer) [104,105,106]. Up to now, prognostic proteomics or glycoproteomic studies have not been performed widely in PDAC patients. Jenkinson and co-workers proposed thrombospondin-1 (TSP-1) as a biomarker for early diagnosis of PDAC. Independent blood samples were collected from patients with PDAC (N = 152), chronic pancreatitis (N = 57), type 2 diabetes (N = 13, benign biliary disease (N = 20), and healthy individuals (N = 56). TSP-1 were generally detected at a lower level as far as 24 months before diagnosis of PDAC. Additionally, low TSP-1 levels were associated with a poor prognosis, making this a potential prognostic marker [107]. Apart from the potential diagnostic value of CEA, which has been researched widely, Boeck and colleagues described CEA as a potential prognostic marker. The CEA levels of 78 PDAC patients were monitored during treatment. Patients were sub-grouped into stable disease (SD), partial response (PR), or progressive disease (PD). The median CEA levels after eight weeks of treatment were 2.6 ng/mL in patients with SD or PR, and 18.1 ng/mL in patients with PD (*p* = 0.002). However, the median decrease of CEA levels in patients with response to therapy was not significantly different from patients with PD (*p* = 0.078). In the same study, cytokeratin-19 was described as a potential prognostic marker for advanced pancreatic cancer. Pre-treatment cytokeratin-19 levels were independently associated with performance status (*p* = 0.04) and disease stage (*p* = 0.0001) [108]. In other studies, the prognostic value of CEA has often been researched mainly using immunoassays. A meta-analysis by Meng and colleagues on 11 studies described an association between high level of CEA and worse overall survival (HR, 1.43; 95% CI, 1.31–1.56) [109]. Other than the above-mentioned studies, studies on cell-free circulating tumor DNA (ctDNA) have been performed using exome sequencing. Additionally, biomarker studies using tissue microarrays and immunochemistry are often performed. These methods do not fall within the scope of this review [110]. Therefore, these studies have been left out of consideration.

### 3.3. Predictive Markers

Biomarkers that provide information on responsiveness to therapy (selective patient treatment) and consequently prevent or terminate unsuccessful treatment regimens with substantial potential for morbidity are not available. Ideally, these markers would also allow monitoring the patient’s response to chemotherapy and provide evidence to guide clinicians in their conversations with patients [111]. So far, only a few predictive protein biomarker studies for PDAC have been performed. SPAN-1, a glycoprotein, emerged as a potential diagnostic marker in 1990 and appeared to be unsuccessful. In 2012, Tsutsumi and colleagues monitored the CA 19-9 and SPAN-1 levels as a potential predictor of tumor progression during chemotherapy. SPAN-1 showed a specificity of 90% prior to treatment. Blood samples and CT scans were performed every four weeks during treatment with gemcitabine in a cohort of 90 PDAC patients. Treatment failure was found in 59% of the patients by using SPAN-1 and 61% for CA 19-9. However, combined with CA19-9, it showed an earlier treatment failure of 72% of all patients with treatment failure, significantly better than CA 19-9 alone (*p* = 0.004) [112]. Several studies have described CA 19-9 as a predictive marker of unresectable disease when its level is high. In a retrospective cohort study consisting of 49 resected PDAC patients and 122 unresected PDAC patients, the CA 19-9 level prior to treatment was evaluated. The CA 19-9 levels were significantly lower in the resected group than in the group with unresectable disease (*p* < 0.001). Additionally, the serum CA 19-9 level accurately predicted resectability in 91% of patients. However, CA 19-9 carries a limit, because patients missing the Lewis group lack secretion of CA 19-9 (5–10% of all patients) [113]. A similar study was performed to assess the preoperative C-reactive protein level to albumin ratio (CAR) as a predictor of overall survival after pancreatic resection for PDAC. In a cohort of 136 patients, with a median follow up of 16.8 months, a high pre-operative CAR appeared to be an independent predictor of poor overall survival (*p* = 0.03) [114]. Further validation and prospective studies of the above-mentioned markers are warranted to determine the clinical value.

## 4. Summary and Conclusions

Despite great efforts in biomarker research, up to this day, only CA 19-9 is FDA approved (Food and Drug Administration) with limited diagnostic value. In this review, current detection and treatment strategies are presented as well as recent protein-based biomarker research, as summarized in a tabular overview (Table 1). From a clinical perspective, there are three main targets for improvement: early detection to provide a window of opportunity where treatment with curative intent is possible; upfront knowledge on individual patient prognosis; and knowledge on response to (chemo)therapy. Prognostic and predictive knowledge can contribute to better therapy selection and work toward personalized medicine. As described throughout this review, recent progress has been made in the discovery of a panel of biomarkers instead of one unimarker. Worldwide, biomarker research is more and more evolving into this approach.

With regard to novel pancreatic cancer biomarkers, there have been significant improvements in both the reported performance of the biomarkers (sensitivity and specificity) and the design of the studies (use of appropriate samples and populations and use of independent training and test samples). Unfortunately, most if not all, candidate biomarker panels have not been confirmed in independent validation sets, especially not in a screening setting. MS technology has matured and robust platforms are now available for quantitative biomarker measurements in discovery studies. These platforms allow the field of proteomics and glycomics to enter the next phase of MS applications by bridging between basic discovery and clinical verification. Further developments are needed to evaluate the performance of a candidate marker when aiming for implementation as a clinical test. For screening of either a high-risk population or an average-risk population for PDAC, blood-based biomarkers require a high specificity for the general population to avoid high numbers of false positives and a high sensitivity for high-risk groups. Several of the protein biomarkers discussed in this manuscript show sensitivities in the range of 50% to 60% at 95% specificity. At a high specificity, which may be required for screening, the reported sensitivities are considerably reduced. So far, protein based prognostic and predictive biomarker research for PDAC has been shown to be limited. For the identification of a potential prognostic or predictive biomarker panel, more research with large patient populations, the use of independent validation sets, and the application of standardized techniques is necessary. 

## Figures and Tables

**Table 1:** Overview of diagnostic, prognostic, and predictive markers.

Marker	Type	Study Size N	Sensitivity (%)	Specificity (%)	Methodology	Reference
CA 19-9 (meta-analysis of 13 studies)	Diagnostic	25–641 PDAC	72–86	68–80	Routine Diagnostics	H. Xing et al.
CEA (meta-analysis of 23 studies)	Diagnostic	17–123 PDAC 15–58 BD	38–50	82–91	Routine Diagnostics	K. Poruk et al.
CA 19-9 combined with: - Apolipoprotein A-IV - Metalloproteinase-1	Diagnostic	42 early stage (I/II) PDAC 72 advanced stage (III/IV) PDAC 31 pancreatitis 35 controls	86	90	Routine Diagnostics & MS-based Proteomics	J. Park et al.
CA 19-9 combined with: - plasma thrombosondin-2	Diagnostic	81 PDAC 80 controls	87	98	Routine Diagnostics & ELISA	J. Kim et al.
CA 19-9 combined with: - Apolipoprotein E - ITIH3 - Apolipoprotein A-I - Apolipoprotein L-1	Diagnostic	80 PDAC 30 BD 40 controls	95	94	Routine Diagnostics & MS-based Proteomics	X. Liu et al.
Peptide signature: - Thrombin light chain - Fibrinogen alpha - Platelet factor 4 - Complement C3f - Factor XIIIa proteins	Diagnostic	Calibration set: 50 PDAC 110 controls Validation set: 39 PDAC 75 controls	78 74	89 91	MS-based Peptidomics	B. Velstra et al. S. Nicolardi et al.
Multimarker panel: - Alpha-2-Glycoprotein - Transthyretin - CA 19-9	Diagnostic	50 PDAC 34 PL 50 controls	82	92	MS-based Proteomics	J. Park et al.
SPAN-1	Diagnostic	64 PDAC 90 other cancers 254 BD 55 controls	81	76	Radioimmuno assay	S. Kiriyama et al.
Multimarker panel: - α -1-antitrypsin - haptoglobin - α-1-acid glycoprotein 1	Diagnostic	154 PDAC 154 controls	Not reported	Not reported	MS-based Proteomics	S. Krishnan et al.
Multimarker panel: - α -1-antichymotrypsin - thrombospondin-1 - haptoglobin	Diagnostic	37 PDAC 30 CP 30 DMII 30 PC 22 OJ 30 controls	91	78	MS-based Proteomics	S. Nie et al.
Thrombospondin-1	Prognostic	152 PDAC 57 CP 13 DMII 20 BD 56 controls	Not reported	Not reported	MS-based Proteomics	Jenkinson et al.
CEA	Prognostic		Not reported	Not reported	Routine Diagnostics	S. Boeck et al.
Cytokeratin-19	Prognostic		Not reported	Not reported	Routine Diagnostics	S. Boeck et al.
SPAN-1	Predictive		90	77	immunoassay	K. Tsutsumi et al.
CA 19-9	Predictive		85	81	Routine Diagnostics	N. Santucci et al.
CAR	Predictive		Not reported	Not reported	Routine Diagnostics	S. Ikuta et al.

CA 19-9: Carbohydrate Antigen 19-9; CEA: Carcinoembryonic Antigen; PDAC: pancreatic ductal adenocarcinoma; ITIH3: inter-alpha trypsin inhibitor heavy chain H3; BD: benign disease; PL: precursor lesions; CP: chronic pancreatitis; DMII: type II diabetes; OJ: obstructive jaundice; CAR: C-reactive protein level to albumin ratio.
